# Intolerance of Uncertainty and Loneliness in Older Adults During the COVID-19 Pandemic

**DOI:** 10.3389/fpsyt.2020.00842

**Published:** 2020-08-19

**Authors:** Eleni Parlapani, Vasiliki Holeva, Vasiliki A. Nikopoulou, Konstantinos Sereslis, Maria Athanasiadou, Athanasios Godosidis, Theano Stephanou, Ioannis Diakogiannis

**Affiliations:** 1st Department of Psychiatry, Faculty of Medicine, Aristotle University of Thessaloniki, Thessaloniki, Greece

**Keywords:** COVID-19, older adults, loneliness, intolerance of uncertainty, depression, anxiety

## Abstract

**Objective:**

The COVID-19 pandemic imposed a psychological burden on people worldwide, including fear and anxiety. Older adults are considered more vulnerable during public health emergency crises. Therefore, the aim of the present study was to investigate the psychological response of older adults during the acute phase of the pandemic in Greece.

**Method:**

This cross-sectional study was part of a larger three-day online survey. A total of 103 participants over the age of 60 fulfilled inclusion criteria. The survey included sociodemographic questions and six psychometric scales: the Fear of COVID-19 Scale (FCV-19S), the Brief Patient Health Questionnaire (PHQ-9) depression scale, the Generalized Anxiety Disorder scale (GAD-7), the Athens Insomnia Scale (AIS), the Intolerance of Uncertainty Scale (IUS-12), and the De Jong Gierveld Loneliness Scale (JGLS).

**Results:**

A significant proportion of the participants reported moderate to severe depressive symptoms (81.6%), moderate to severe anxiety symptoms (84.5%), as well as disrupted sleep (37.9%). Women reported significantly higher levels of COVID-19–related fear, more severe depressive symptoms and sleep disturbances, as well as higher levels of intolerance of uncertainty. Participants living alone showed higher levels of loneliness. Intolerance of uncertainty was shown to modulate levels of loneliness.

**Conclusions:**

During the quarantine, attention was promptly drawn upon the risks related with older people’s loneliness. Studies identifying factors that may contribute to loneliness during a public health emergency facilitate the implementation of supportive interventions. Preparedness to address and manage older people’s loneliness may limit this deleterious emotional response during the pandemic, as well as at the post-COVID-19 phase.

## Introduction

The World Health Organization (WHO) declared COVID-19, the disease associated with the novel “Severe Acute Respiratory Syndrome Coronavirus 2” SARS-CoV-2, a “Public Health Emergency of International Concern” on January 30 (1), and a “pandemic” on March 11, 2020 (2). In Greece, the first confirmed COVID-19 case was reported on February 26. While the number of COVID-19 positive cases was constantly increasing, restriction measures were stepwise introduced. After 695 COVID-19 confirmed cases and 17 COVID-19–related deaths had been reported, a 6-week national lockdown was imposed on March 23 (3).

The COVID-19 pandemic induced worry (4), fear (5), anxiety, and depressive symptoms (6), as well as insomnia (7). Older adults are considered more vulnerable during public emergency crises (8). Their vulnerability is linked with the age-related compromised physical state, increased prevalence of chronic health conditions and other disabilities, cognitive abilities’ decline, as well as the potential presence of adverse psychosocial conditions (9). Similarly, the COVID-19 pandemic affected older people in many different aspects. Fear of contracting the virus and fear of death impinged on older people (10), since increased age is a risk factor for severe disease due to compromised immune system function and the higher prevalence of risk conditions for severe COVID-19, such as hypertension, diabetes mellitus, cardiovascular, and respiratory diseases (11); around 66% of people over the age of 70 were shown to suffer from at least one chronic medical condition (12). The case fatality ratio was estimated at 1.4% for people under the age of 60, at 4.5% for people over 60, whereas at 13.4 for people over 80 (13). During the pandemic, around 95% of COVID-19–related deaths in Europe, 80% of fatal COVID-19 cases in the United States, and 80% of fatal cases in China involved patients over the age of 60 to 65 (14). By June 1, 2020, 76.5% of the 179 COVID-19–related deaths in Greece involved patients over the age of 65 (15). Despite the emphasis placed by WHO on the older residents of long-term care facilities (16), a great number of COVID-19–related deaths was reported in care homes in countries severely affected by the pandemic. Although official records were not always complete and accurate, available data suggested that between the middle of April and the beginning of May, 67% of total COVID-19–related deaths in Spain and 37% of total COVID-19–related deaths in France involved residents in care homes; death numbers in care homes in the United Kingdom were the greatest since 1993 (17), while roughly one out of five COVID-19–related deaths in the United States was recorded in nursing homes (14).

The older high-risk group for severe COVID-19 illness was also in danger of having to cope with ageism, a term coined by Dr. Robert Butler to broach the matter of discrimination against older people and the common use of stereotypes (18), since ageism may involve age discrimination in health care as well (19). During the pandemic and in face of medical equipment shortage, age was a criterion that may have been applied in ventilator triage policies, in such “if patients have similar expected incremental increases in survival, triage decisions may include consideration of patient age based on the principle that people should have the opportunity to live as much of the normal human life cycle as possible”; “in the event that there are ties in priority scores between patients, life-cycle considerations will be used as a tiebreaker, with priority going to younger patients, who have had less opportunity to live through life-stages” (20). Despite criticism against such policies (21), healthcare professionals in countries severely affected by the pandemic were forced to prioritize younger over older patients due to the healthcare system’s overload with COVID-19 patients (22).

Furthermore, measures to preserve resources for the management of the pandemic, such as suspension and/or postponement of health services for non-emergent conditions unrelated to COVID-19 (23), posed a risk to older people’s physical health (10), since older adults are more likely to suffer from chronic conditions requiring regular doctor visits and long-term medication (14). Similarly to other countries, the guidelines by the Hellenic National Public Health Organization (24) to restrict virus spread in hospitals included canceling all non-emergent outpatients’ visits and surgical procedures. In addition, fear of retracting the virus may have been associated with decreased hospital visits and hospitalizations for other conditions. Although there have been no official data on hospital visits at the emergency departments for COVID-19–unrelated reasons, there were anecdotal records of markedly decreased visit numbers in all departments (25). Altogether, older people’s chronic health issues were in danger of being lower-prioritized, due to the necessity of placing emphasis on containing the pandemic (26).

A pandemic is a worldwide health emergency crisis associated with fear (27), an “emotional reflex” related with collective memories of former deadly infectious diseases (28). Fear of the unknown (29) and worry (30) are emotions related to Intolerance of Uncertainty (IU), a characteristic originally conceptualized as the cognitive, emotional, and behavioral responses to uncertainty in everyday situations (29, 31). Throughout the years, researchers provided more definitions, in an effort to describe this concept more accurately (29). Individuals with high IU consider the possibility of a negative event as unacceptable and threatening (32), are prone to worry about unpredictable, future negative events and tend to perceive uncertain and ambiguous situations as threatening (33). Two dimensions were incorporated in the concept of IU, prospective and inhibitory IU (34); prospective IU represents the cognitive dimension, that is, cognitive assessments of threat related with unforeseeable events and desire for foreseeable events; inhibitory IU represents the behavioral dimension, that is, behavioral inhibition or “paralysis” due to uncertainty (35). Lately, IU has been conceptualized as an individual feature, a trait, reflecting negative beliefs about uncertainty and, according to Carleton (36), the incapacity to bear the response “triggered by the perceived absence of salient, key, or sufficient information”. This tendency toward negative perceptions and responses to uncertain circumstances was associated with worry (37) and anxiety-related disorders (33). On the other side, “state” IU may also emerge in response to uncertain stimuli, on the ground of high or normal trait IU, or as part of emotional disorders (33) that may have emerged during the COVID-19 pandemic (38). Moreover, IU was found to be a predictor of COVID-19–related fear (39).

Social-physical distancing and quarantine, the main strategies implemented to prevent the spread of COVID-19 (40), were related with psychological distress, depression, anxiety, insomnia, and social detachment (41). The latter imposed a great psychological burden particularly on dependent older people living alone and/or receiving home care by family members, friends, caregivers or social services. Although prompted by empathy and fear for the high-risk community members’ safety (42), physical distancing was associated with reduced home visits, disruption of regular care provision, and focus on only basic needs. Still, the fragile health condition of very old people may be affected by inadequate nutrition, lack of personal and home hygiene, restriction of physical exercise, and irregular supervision of medication intake. Moreover, lack of social contacts contributes to cognitive decline, which, in turn, may lead to risky behavioral disturbances (25). In addition, common socialization channels for older people, such as meeting centers and churches, were locked down. As a result, restriction measures deprived older adults of the opportunity to socialize with their peers, compromising psychological well-being by bringing on isolation, a condition posing a great risk for depression, anxiety (43), as well as loneliness (10).

“Loneliness” is a term encompassing a wide range of definitions, among which, “a subjective perception of a negative emotional state related with the divergence between desired and existing relations with others” (44). According to Weiss (45), loneliness may be emotional or social. Emotional loneliness, a subjective experience, is related with the absence of a desirable close and affectionate bonding with a person, absence of someone to turn to. Social loneliness, an objective condition, involves lack of contacts, social networks and the sense of belonging to a smaller or wider circle of people. Therefore, the term “loneliness” encompasses both qualitative and quantitative aspects of relationships (46, 47). In older adults, loneliness was related with depression, anxiety, increased risk of further social dysconnectivity (48), poor global sleep satisfaction (49), and deterioration of cognitive functions (50). Moreover, it was observed that lonely older adults engage in unhealthy practices, such as smoking, alcohol consumption, and less physical activity, which compromise physical health (44, 51); loneliness was associated with increased risk of coronary heart disease and deterioration of cardiovascular diseases (52), a well acknowledged risk factor for severe COVID-19 (53). Altogether, loneliness was shown to have an impact on older people’s mental health, physical health and overall well-being (44, 51). Therefore, loneliness remains an issue of significant research interest in older adults.

In 2018, people over the age of 65 represented one fifth of the European Union population, an increase of 2.6% compared with 10 years earlier. Greece offered the second highest share of people over 65 years in the total population (21.8%) after Italy (22.6%). In 2018, the old-age dependency ratio (OADR; an index used to investigate the level of support offered to older people by the working population, defined as the number of old-age dependents over the age of 65 per 100 persons of working ages 20–64) was estimated at 34.1% in Greece, that is, around three working age people for every person aged over 65 (54); in 2019, the OADR raised at 37% and is expected to reach a 75% by 2050, placing Greece within the 10 countries with the highest OADR worldwide (55).

Altogether, the population is ageing all over the world, a “longevity revolution”. By 2050, one out of six individuals worldwide will exceed the age of 65, compared with 2019 data indicating that 1 out of 11 exceeded the age of 65. People will have a 90% chance of surviving up to the age of 65 in countries with high life expectancy. In most developed countries, the proportion of older adult life will correspond to one quarter of total life time (55). Moreover, the chronological age may not always be identical with the biological age (56). According to the latest Eurostat data, women and men at the age of 65 are expected to live an average of 9.5 years in good health. Specifically, in Greece, both women and men at the age of 65 are expected to live in good health until the age of 72.7 (57). Since health expectancy has been prolonged, older people may remain active and contribute to the family and societal life in multiple manners. During the COVID-19 pandemic, retired health professionals were called upon to support the overloaded healthcare system in many countries, including Italy (58), Spain (59), the United Kingdom (60), and the United States (61).

Taking into account that older adults comprise a significant proportion of the population, may continue to retain an active role in society (62), and may be more vulnerable during public health emergencies (9), older adults remain a significant research population. Therefore, this study focused on an older Greek population during the COVID-19 crisis. Taking available literature into account, the study aimed to investigate the psychological impact of COVID-19, that is, fear, depressive and anxiety symptoms, as well as sleep disturbances, on older individuals. Furthermore, the study focused on loneliness during the COVID-19 pandemic, and investigated whether fear of COVID-19, depressive and anxiety symptoms, insomnia, and IU were potential predictors of loneliness.

## Methods

### Study Population and Design

A non-standard, though widely accepted cutoff threshold to define an older population in developed countries is the age of 60. The definition of “old” is also related with one’s employment-retirement status; in the majority of countries the retirement age ranges from 60 to 65 years (63). In Greece, three out of four employees retire by the age of 61 (64). Taken together, the present study included older adults over the age of 60.

This cross-sectional study was part of a larger online survey (3,029 participants) targeting the Greek general population. The survey, created via Qualtrics online survey software (65), was distributed through the social media and was available online for a period of three days, three weeks after a national lockdown had been imposed in Greece. Information about the study’s scope and usefulness was provided in the survey’s homepage. Before taking the survey, respondents were requested to formally consent to their participation. Acceptance to participate was a prerequisite for study inclusion. Participation was voluntary and anonymous.

Initially, 120 consenting participants fulfilling the age criterion completed the survey (3.96% of the original sample). Among these, 17 (5 males and 12 females) reported that they suffered from a pre-existing psychiatric disorder during the last 6 months, for which they received psychiatric medication (including antidepressants, antipsychotics, tranquilizers, and hypnotics). These participants were excluded from the analysis. As a result, a total of 103 participants (3.4% of the original sample) entered the study.

Ethical approval was received from the Scientific Committee of the General Hospital “Papageorgiou” Review Board.

### Measures

At first, the survey included basic sociodemographic questions, including age, gender, residential area, living status, and educational level. Consequently, respondents completed the following psychometric scales:

1. The Greek version of the Fear of COVID-19 Scale (FCV-19S) (38, 66). The scale is a reliable and valid unidimensional self-report tool, recently developed to facilitate research during the COVID-19 pandemic. The scale assesses COVID-19–related fear independent of gender and age. It consists of seven items, e.g., item 1, “I am most afraid of coronavirus-19”; item 4, “I am afraid of losing my life because of coronavirus-19”; item 7, “My heart races or palpitates when I think about getting coronavirus-19”. Each item is rated on a 5-point Likert-type scale as follows: 1 = strongly disagree; 2 = disagree; 3 = neither agree nor disagree; 4 = agree; 5 = strongly agree. The total score ranges between 7 and 35. Higher scores reflect greater fear of COVID-19.
2. The Greek version of the Brief Patient Health Questionnaire (PHQ-9) depression scale (67, 68). The scale constitutes the 9-item depression module from the complete Patient Health Questionnaire (PHQ). It is a self-report tool used for the diagnosis of major depression and subthreshold depression in the general population (69), assessing depressive symptoms’ severity over the past two weeks. Each of the nine items (e.g., item 1, “Little interest or pleasure in doing things”) is rated on a 4-point severity scale (0 = not at all; 1 = several days; 2 = more than half the days; 3 = nearly every day). The total score ranges between 0 and 27. Symptoms’ severity is assessed based on the following cutoff scores: 0–4 = minimal or none; 5–9 = mild; 10–14 = moderate; 15–19 = moderately severe; 20–27 = severe (the cutoff point of 10 or greater may indicate a clinically significant condition).
  The last item of PHQ-9 exploring suicidal ideation (item 9: Over the last two weeks, how often have you been bothered by thoughts that you would be better off dead or of hurting yourself in some way?) was shown to be a strong predictor of suicide attempts regardless of age (70), and was therefore separately analyzed (item 9 score > 0) to investigate the prevalence of suicidal ideation in the present sample.
3. The Greek version of the Generalized Anxiety Disorder scale (GAD-7) (71, 72). The scale was proven a useful self-administered tool for the assessment of anxiety symptoms’ severity over the past two weeks. Each of the seven items (e.g., item 1, “Feeling nervous, anxious or on edge”) is rated on a 4-point severity scale (0 = not at all; 1 = several days; 2 = more than half the days; 3 = nearly every day). The total score ranges between 0 and 21. Symptoms’ severity is assessed based on the following cutoff scores: 0–5 = mild; 6–10 = moderate; 11–15 = moderately severe; 15–21 = severe (the cutoff point of 10 or greater may indicate a clinically significant condition).
4. The Athens Insomnia Scale (AIS) (73). The scale is an 8-item instrument originally developed in Greek to evaluate sleep duration and quality according to the International Classification of Diseases, 10th Revision, criteria. The first five items explore sleep induction, awakenings during the night, final awakening, total sleep duration, and sleep quality, while the last three items explore day-time well-being, physical and mental functioning, as well as sleepiness. Each item is rated on a 4-point severity scale ranging from 0 (no considerable sleep disturbances) to 4 (serious/intense sleep disturbances). The total score ranges from 0 to 32; higher scores reflect more severe sleep difficulties. The cutoff score of 10 was proposed for usage in the general population (positive predictive value of about 90%) and was applied in this study to distinguish non-insomniacs from insomniacs (74).
5. The Greek version of Intolerance of Uncertainty Scale (IUS-12) (35, 75). The scale is a 12-item instrument, derived from the original 27-item IU questionnaire (31). It assesses reactions to ambiguous conditions, uncertainty and forthcoming events. The scale displayed strong psychometric properties, was accepted as a transdiagnostic assessment tool for trait IU (76), and demonstrated a two-factor structure, evaluating prospective IU (7-item subscale; sum of items 1, 2, 4, 5, 8, 9, and 11; e.g., item 1: “Unforeseen events upset me greatly”), and inhibitory IU, related with avoidance (5-item subscale; sum of items 3, 6, 7, 10, and 12; e.g., item 3: “Uncertainty keeps me from living a full life”). Each item is rated on a 5-point Likert-type scale ranging from 1 (not at all characteristic of me) to 5 (entirely characteristic of me). The total score ranges between 12 and 60. Higher scores indicate greater levels of IU. The Greek version’s Confirmatory Factor Analysis resulted in the following parameters: chi-square goodness of fit test = χ^2^ (54) = 1176.40, p <.001, RMSEA = 0.09, 90% CI = [0.08, 0.09], CFI = 0.86, TLI = 0.83, and SRMR = 0.05. Convergent validity was established by correlating IUS-12 with GAD-7 [(r_p_ = 0.58, p <.001, 95% CI (0.56, 0.61)]. The items for IUS-12 had a Cronbach’s alpha coefficient based on standardized items of 0.90.
6. The Greek version of the De Jong Gierveld Loneliness Scale (JGLS) (46, 77). This is a 6-item measure, the short version of the original 11-item De Jong Gierveld Loneliness Scale (78), consisting of two subscales, a 3-item subscale assessing emotional loneliness (e.g., item 1, “I experience a general sense of emptiness”) and a 3-item subscale assessing social loneliness (e.g., item 4, “There are plenty of people I can rely on when I have problems”). Each question may be answered with “yes”, “more or less” or “no”. To rate the items, the “more or less” and “yes” answers are scored with one on the negatively worded questions, that is, items 1, 2, and 3 assessing emotional loneliness. On the contrary, on the positively worded items, that is, items 4, 5, and 6 assessing social loneliness, the “more or less” and “no” answers are scored with one. The total score for both emotional and social loneliness ranges from 0 to 3; the total loneliness score ranges from 0 (least lonely) to 6 (most lonely). The Greek version’s Confirmatory Factor Analysis resulted in the following parameters: chi-square goodness of fit test = χ^2^ (9) = 20.04, p = .018, RMSEA = 0.12, 90% CI = [0.05, 0.19], CFI = 0.91, TLI = 0.85 and SRMR = 0.08. Convergent validity was established by correlating JGLS with PHQ-9 [(r_p_ = 0.31, p <.001, 95% CI (0.12, 0.47)]. The items for JGLS had a Cronbach’s alpha coefficient based on standardized items of 0.70.

#### Data Analysis

Data and parameter estimates were presented as numbers (N) and percentages (%) or as mean values (M) and standard deviations (SD). Independent samples t-tests and one-way analyses of variance (ANOVA), with Bonferroni Correction were performed to explore participants’ differences regarding the main psychometric scales. Chi-squared cross-tabulation was used to identify significant differences among the severity categories of fear, anxiety, and depression.

Linear regression analysis was performed to calculate the associations of loneliness (dependent variable) with IU, depressive and anxiety symptoms (independent variables).

Statistical analyses were performed by the IBM Statistical Package for Social Sciences (SPSS), Version 26.0.

## Results

The study included 40 male and 63 female participants. The majority of survey respondents were urban residents (80.6%), lived together with their family or a caregiver (78.6%) and had a university degree (45.6%) (**Table 1**).

**Table 1:** Participants’ sociodemographic and clinical characteristics.

Sociodemographic characteristics	Overall (n = 103)		Male (n = 40)		Female (n = 63)			
Age	M	SD	M	SD	M	SD	p	
	69.85	5.26	70.87	5.84	69.2	4.79	>.001	
Residential area	N	%	N	%	N	%	p	
Urban	83	80.6	31	79.5	52	83.9	>.001	
Small City	5	4.9	1	2.6	4	6.5		
Rural	13	12.6	7	17.9	6	9.7		
Living with	N	%	N	%	N	%	p	
Family/caregiver	81	78.6	33	82.5	48	76.2	>.001	
Alone	20	19.4	6	15	14	22.2		
Education	N	%	N	%	N	%	p	
Elementary school	6	5.8	2	5.3	4	6.3	>.001	
Middle school	2	1.9	0	0	2	3.2		
High school	25	24.3	13	34.2	12	19		
University	47	45.6	14	36.8	33	52.4		
MSc	14	13.6	6	15.8	8	12.7		
PhD	7	6.8	3	7.9	4	6.3		
Clinical characteristics	M	SD	M	SD	M	SD	t-test	Cohen’s d
FCV-19S	18.48	5.32	16.75	5.43	19.54	5.01	t = −2.551, df = 93, p = .012	.53
PHQ-9	13.68	4.22	12.45	3.74	14.46	4.35	t = −2.407, df = 101, p = .018	.65
GAD-7	13.21	4.67	12.61	4.83	13.59	4.58	p > .001	
AIS	12.79	3.84	11.83	3.44	13.40	43.99	t = −2.05, df = 101, p = .043	.041
IUS-12	31.75	8.58	29.33	7.79	33.29	8.76	t = −2.331, df = 101, p = .022	.54
JGLS	2.35	1.64	2.23	1.57	2.43	1.70	p > .001	

FCV-19S, Fear of COVID-19 Scale; PHQ-9, Brief Patient Health Questionnaire Depression Scale; GAD-7, Generalized Anxiety Disorder scale; AIS, Athens Insomnia Scale; IUS-12, Intolerance of Uncertainty Scale; JGLS, De Jong Gierveld Loneliness Scale; M, mean; SD, Standard Deviation.

Females reported significantly higher levels of COVID-19–related fear (p = .012), more severe depressive symptoms (p = .018) and more severe sleep disturbances (p = .043). Furthermore, females showed higher levels of IU (p = .022) compared with males. Specifically, females showed higher levels of prospective IU (M = 19.73, SD = 4.95) compared with males (M = 16.97, SD = 4.72), and this difference was statistically significant (t = −2.759, df = 97, p = .007). Although females showed higher levels of inhibitory IU (M = 14.07, SD = 4.36) than males (M = 12.64, SD = 3.70), this difference was not statistically significant (t = −1.686, df = 98, p = .95). Lastly, females and males did not differ with regard to anxiety symptoms’ severity and loneliness (**Table 1**).

Participants living alone showed higher levels of loneliness (p = .004) compared with participants living together with their family or a caregiver. On the contrary, there were no statistically significant differences in the levels of COVID-19–related fear, depressive and anxiety symptoms’ severity, sleep difficulties, as well as IU between participants living alone and participants living together with their family or a caregiver (**Table 2**).

**Table 2:** Clinical characteristics in relation with living status.

Clinical characteristics	Living with				t-test		Cohen’s d
	Family/caregiver		Alone				
	M	SD	M	SD		
FCV-19S	18.65	5.37	17.94	5.37	t_(92)_ = −.480, p = .606		–
PHQ-9	13.47	3.74	14.60	5.36	t_(100)_ = 1.064, p = .245		–
GAD-7	13.32	4.66	12.56	5.09	t_(91)_ = −.578, p = .502		–
AIS	12.67	3.75	13.35	4.40	t_(99)_ =.703, df=99, p=.483		–
IUS-12	31.49	8.54	32.70	8.99	t_(100)_ = .560, p = .620		–
JGLS	2.09	1.55	3.25	1.71	t_(99)_ = 2.932, p = .004		.71

FCV-19S, Fear of COVID-19 Scale; PHQ-9, Brief Patient Health Questionnaire Depression Scale; GAD-7, Generalized Anxiety Disorder scale; AIS, Athens Insomnia Scale; IUS-12, Intolerance of Uncertainty Scale; JGLS, De Jong Gierveld Loneliness Scale; M, mean; SD, Standard Deviation.

A significant proportion of the participants reported moderate to severe depressive symptoms (81.6%), moderate to severe anxiety symptoms (84.5%), as well as disrupted sleep (37.9%) (**Table 3**). Moreover, a total of 35 participants (33.9%; 12 males and 23 females) reported suicidal ideation based on PHQ-9 item 9 (score > 0), while 70% of the male and 63.5% of the female participants did not report any suicidal thoughts.

**Table 3:** Participants’ grouping according to psychometric scales’ cutoff scores.

	Overall		Male		Female		x^2^	df	p	Vcramer
	N	%	N	%	N	%				
Depressive symptoms										
Mild	18	17.5	10	25	8	12.9	4.604	3	.203	.203
Moderate	45	43.7	18	45	27	43.5				
Moderately severe	31	30.1	11	27.5	20	32.3				
Severe	8	7.8	1	2.5	7	11.3				
Total	102	99.1	40	100	62	100				
Anxiety symptoms										
Mild	3	2.9	2	5.9	1	1.8	3.91	3	.270	.270
Moderate	28	28.2	11	32.4	17	30.9				
Moderately severe	33	32	15	44.1	18	32.7				
Severe	25	24.3	6	17.6	19	34.5				
Total	89	87.4	34	100	55	100				
Insomnia										
Absent	64	62.1	30	75	34	54.0	4.6	1	.038	.032
Present	39	37.9	10	25	29	46.0				
Total	103	100	40	100	63	100				

Linear regression analysis was performed to identify significant predictors of loneliness. AIS and FCV-19S did not enter the model as their correlation with JGLS was non-significant (p >.05). The highest correlation of JGLS was with IUS-12 (r = .335, p <.01) and the lowest with anxiety (r = .263, p <.05). All needed transformation was completed before the analysis and relevant statistical assumptions were met.

The results of the analysis revealed that the linear combination of IUS-12, PHQ-9, and GAD-7 accounted for a significant amount of variance of loneliness [R^2^ = 0.14, F(3,89) = 4.93, p = .003]. Further examination of the beta weights on each scale indicated that IUS-12 score significantly predicted JGLS score [B = 0.05, t(89) = 2.33, p = .022)]. On the contrary, PHQ-9 and GAD-7 scores failed to present significance as predictors of the JGLS score [B = 0.04, t(89) = 0.71, p = .482; B = 0.02, t(89) = 0.28, p = .778] (**Table 4**).

**Table 4:** Linear regression with IUS-12, PHQ-9, and GAD-7 predicting JGLS.

Variable	B	SE	95% CI	β	t	p
(Intercept)	−0.16	0.67	[−1.49, 1.16]	0.00	−0.24	.809
IUS-12	0.05	0.02	[0.01, 0.10]	0.28	2.33	.022
PHQ-9	0.04	0.06	[−0.08, 0.17]	0.11	0.71	.482
GAD-7	0.02	0.06	[−0.10, 0.13]	0.04	0.28	.778

IUS-12, Intolerance of Uncertainty Scale; PHQ-9, Brief Patient Health Questionnaire Depression Scale; GAD-7, Generalized Anxiety Disorder scale; JGLS, De Jong Gierveld Loneliness Scale.

## Discussion

The awareness that increased age is a risk factor for COVID-19–related mortality, together with the restriction of family and social contacts due to quarantine measures, had a psychological impact on older adults during the pandemic (79). Although a study of a Chinese population reported that adults over the age of 60 displayed the highest COVID-19 peritraumatic distress index (80), other studies of different Chinese populations showed that the prevalence of posttraumatic stress symptoms (81) and the severity of depressive and anxiety symptoms (6) were not differentiated based on age. Moreover, a study of a Spanish population observed that adults over the age of 65 reported less severe depressive and anxiety symptoms compared with younger adults under the age of 35 (82). Altogether, further research is required to explore the differences in the psychological impact of COVID-19 between younger and older adults.

According to previous community-based studies, published between 2005 and 2018, the prevalence of moderate to severe depressive symptoms in Greek adults over the age of 60 ranged from 30% to 46% (83–89), depending on sample size and differences in study groups and assessment methods. This study showed that roughly 8 out of 10 older adults reported moderate to severe depressive and anxiety symptoms. Therefore, current results indicated that during the COVID-19 pandemic, the prevalence of depressive/anxiety symptoms may have increased. Furthermore, around 3 out of 10 participants reported insomnia.

Greece continues to belong among the countries with the lowest suicide rates (5 suicide deaths/100,000 population in a year versus an average suicide rate of 11.3 in European Union countries in 2014) (90, 91). It has been suggested that suicide rates may increase during the COVID-19 pandemic (92). Older adults, especially the ones suffering from depression, may be more vulnerable to suicide during a health crisis (93). According to current results, 34% of the participants reported suicidal ideation, based on the last PHQ-9 item, a finding potentially reflecting the pressure experienced during the imposed lockdown.

There was evidence that the psychological impact of COVID-19 was greater in women compared with men, that is, women expressed more worry (4) and showed more severe depression, anxiety (6), psychological distress (80), and insomnia (7). Based on current results, older women showed significantly higher levels of COVID-19–related fear, more severe depressive symptoms and greater sleep difficulties compared with older men. On the contrary, severity of anxiety symptoms was not differentiated based on gender. Therefore, it may be postulated that although older women were shown to report altogether more worry, as well as more severe depressive and anxiety symptoms compared with older men (94), the novel COVID-19 circumstances imposed similar levels of anxiety on both genders.

This study also explored IU in older individuals, using a gender invariant scale (95). According to the results, women showed higher levels of IU compared with men; this difference was particularly significant with regard to prospective IU, reflecting more cognitive assessments of threat regarding unforeseeable events and more desire for predictability (35), a finding related with the fact that women tend to worry more than men (96). Still, there is only limited information about gender differences in IU, suggesting that although women tend to worry more than men, IU levels are not significantly different based on gender (97). There is also limited evidence that individuals over the age of 65 show lower levels of IU compared with younger individuals (98), supporting the theory that ageing may modify personality characteristics (99). Older people’s better emotional regulation and maturation through long-term experience with unforeseeable and ambiguous situations may attenuate trait IU, alleviating worry in older ages (98). Still, to the best of our knowledge, gender-related differences in IU in older individuals have not been reported yet. Further research of IU in older women and men is warranted, since it was suggested that IU constitutes a transdiagnostic mechanism contributing to a variety of psychological symptoms, with a more pronounced involvement in the manifestation of anxiety and depressive symptoms (100). Moreover, during the COVID-19 pandemic, IU was related with higher fear of COVID-19 (39), insomnia (7), and less positivity (101).

Anecdotal statements of gradually increasing loneliness in older people over the past decades were not supported by longitudinal studies. Becoming older is misguidedly identified as becoming lonelier. Loneliness affects younger adults as well. The highest prevalence of loneliness was observed over the age of 80 (47), while loneliness was shown to increase with age only over the age of 80 (102). Therefore, the relatively low loneliness levels observed in this study may be explained by the sample’s lower mean age. Moreover, old age alone is not a sufficient condition for the manifestation of loneliness, since there are more contributors to loneliness, such as not living together with a spouse/partner and limited socialization (103).

Evidence of gender differences in loneliness is inconclusive. Reports of increased loneliness in women compared with men were provided by studies using another tool, the UCLA loneliness scale, or one item indicators, and not the DeJong-Gierveld scale, applied in this study. Moreover, gender alone may not be an independent factor predicting loneliness in older individuals (102–104). Similarly, the current results did not support gender-related differences in loneliness.

Attention was promptly drawn upon the risks related with older people’s social isolation during the quarantine (10). The magnitude of the pandemic’s psychological impact on older adults is related with sociocultural factors mediating older people’s family and social connectedness (105, 106). According to Reher’s work (2004), the center and north of Europe was characterized by weaker, while the Mediterranean by stronger family ties (107). The grade of familialism was shown to increase from North to South Europe; Greece was shown to be a country with strong familistic attitudes toward older people compared with other European countries (108). The living status followed the same “North to South” pattern, that is, the proportion of older people living alone was lower in South Europe (109). According to Eurostat, an average of 32.1% of older adults in Europe live alone, whereas in Greece, only about one out of four older adults lives alone (57) Although living alone does not necessarily equate loneliness (110), it was proven a strong predictor of loneliness (111). Similarly, this study showed that living alone was related with higher levels of loneliness in older adults.

Furthermore, having children (104), as well as being a member of a joint family were related with less loneliness, since “family” offers older people security, comfort, connectedness, and support. Loneliness was not shown to be a major issue for older members of an extended family, being collectively taken care of by other family members (112). The strong family bonds in Greece date back to the “Golden Age” of Pericles (fifth century BC). In Ancient Greece, “geroboskia” or “gerotrophia”, that is, providing care for older people, was a sacred duty performed by family members. Moreover, severe penalties were imposed on offspring refusing to provide care for their older parents. As a result, at that time, there were no public facilities for the care of older people (113). Ancient Greeks’ practices toward older people were a legacy to the next generations. During the following centuries, family members remained the traditional caregivers for older people in Greece. Moreover, in the beginning of the 20^th^ century, Greek families were organized in an extended form, not only embracing older family members, but also placing them on top of the family hierarchy. Patriarchal authority exercised by older males involved decisions on financial matters and the future spouses of children and grandchildren, while matriarchal authority exercised by older women involved organization of housework. Lately, the development of nuclear families disempowered older people, weakening their position in society (114). However, strong bonds between the younger and the older family members are maintained. Residential proximity is often pursued between parents and at least one of the adult children. The strong family values render “family” a core component of the Greek society. Altogether, the Greek society is still governed by a moral duty toward its older members. Moreover, the article 1485 of the Greek Civil Code imposes a legal duty as well, obligating adult children to take care of their parents (115).

Although depression and anxiety were shown to contribute to loneliness (48), the current results highlighted the modulating effect of IU on severity of loneliness. This study was conducted three weeks after a national lockdown had been imposed in Greece. The family network remains a cornerstone in the care and welfare of older adults in Greece. Uncertainty about the duration of the quarantine and the necessity to maintain physical distancing from family and friends may have intensified loneliness. The fact that the Greek sociocultural background nurtures the moral obligation to provide support and emotional care to older people may elevate older Greeks’ expectations and needs from their family. Therefore, loneliness may be easier to experience, when expectations are not fully met (47). In addition, older adults support their adult children in everyday routine. Grandparents in Greece take care of their grandchildren to facilitate working mothers (115). Caring for a grandchild was shown to expand older people’s social network and to reduce loneliness (116, 117). Restriction measures and isolation deprived older people of the opportunity to contribute to their family and therefore to retain the sense of a significant societal role and connectedness. Families kept their older members in safety, away from the virus, and managed alone. This new situation may have raised older people’s uncertainty about the importance of their family role and their societal position, contributing to loneliness. Lastly, restriction measures compelled older people to become more involved in technology. Older people are more reluctant with the Internet use. In Greece, only about 4% of people within the age range of 65–74 use the Internet (118). The necessity to get acquainted with the Internet technology and to develop new skills, for instance use of online bill pay, potentially raised older people’s uncertainty. The need to undertake new responsibilities may have led to a sense that instead of being taken care of, older people were left to manage on their own.

To the best of our knowledge, up to date there have been no published studies of older adults during the COVID-19 crisis in Greece. This study investigated the psychological impact of COVID-19 on older people during the acute phase of the pandemic. According to the results, the majority of study participants manifested moderate to severe depressive and anxiety symptoms, women carried a heavier psychological burden, and intolerance of uncertainty modulated loneliness severity. Studies identifying factors that may have contributed to loneliness during the COVID-19 pandemic facilitate the implementation of supportive interventions. Older individuals show a preference for goals and environments with minimal negative emotional load, that is, a protective, “stable” surrounding, alleviating uncertainty (119). Restriction measures and disruption of daily routine was a significant source of uncertainty during the COVID-19 pandemic. Therefore, any form of regular care, such as delivering groceries and medical supplies to older people regardless of their ability to provide for themselves or not, signifies care and ensures brief, but frequent meetings. This approach restores some daily routine, mitigates uncertainty, and may therefore alleviate related feelings of loneliness. Limiting exposure to information overload by the media is another remedy to relief uncertainty (10). Introducing older people to online technology enhances social contacts (120), while frequent telephone contacts and involvement of older people in decision-making about family matters nurture a sense of connectedness, which was shown to promote older adults’ well-being during the previous SARS outbreak in 2003 (121). Among a variety of other policies and programs (21), the initiative taken by the Doctors of the World/Médecins du Monde-Greece to support isolated older adults over the age of 60 (122), as well as various national telephone psychosocial support services aimed to provide assistance and psychological care to older Greeks in need during the pandemic.

Still, the present study had some limitations. The cross-sectional design did not allow investigation of causal relationships. Results were based on self-report tools, and may therefore suffer from bias. Moreover, despite the attempts to focus respondents’ attention on the COVID-19–related impact (the survey’s headline was “The psychological burden related with the COVID-19 pandemic crisis”; the survey’s homepage included a description of the study’s scope), and although participants with pre-existing psychiatric disorders were excluded from the analysis, it cannot be ruled out that study results may have reflected, at least partially, pre-existing psychological symptoms. Furthermore, due to the strict restriction measures, the study was conducted through an online survey distributed by the social media, which are used only by 2.3–5.5% of adults over the age of 65 in Greece (123). Consequently, the sample was relatively small, while less educated and socially disadvantaged older adults may not have been adequately represented. Lastly, online surveys suffer from the so-called “volunteer-effect”. Therefore, responders’ characteristics may differ substantially from non-responders, limiting results’ generalizability (124).

Conclusively, the COVID-19 pandemic crisis unveiled a lack of sufficient data on the older population (14), a significant proportion of the total population in many countries that should not be overlooked. Healthy ageing does not solely involve physical health attainment, but also nurture of psychological resources (125). This crisis may offer the opportunity to address issues related with more efficient care for older adults during public health crises (21). As a result, awareness and therefore preparedness to assess and address loneliness in older adults may rise during the post-pandemic period, allowing the development of management strategies to eliminate this deleterious emotional response (126).

## Data Availability Statement

The raw data supporting the conclusions of this article will be made available by the authors, without undue reservation.

## Ethics Statement

This study involving human participants was reviewed by the Scientific Committee of the General Hospital “Papageorgiou” Review Board, Thessaloniki, Greece. Ethical approval was received prior to data collection. Before entering the online-survey, respondents were requested to indicate their consent. The study was anonymous.

## Author Contributions

EP and VH contributed equally to this study. EP contributed to intellectual input and data interpretation, and wrote the first draft of the manuscript. VH contributed to study’s conception and design, as well as to data management interpretation. VN contributed to data management and literature search. KS, MA, AG, and TS contributed to literature search and paper editing. ID supervised the study and contributed to the final revision of the manuscript. All authors contributed to the article and approved the submitted version.

## Conflict of Interest

The authors declare that the research was conducted in the absence of any commercial or financial relationships that could be construed as a potential conflict of interest.
